# How much is too much: A case study of local self-government units in Slovakia using absolute variability to determine the importance of financial criteria in MCDM analysis

**DOI:** 10.1371/journal.pone.0311842

**Published:** 2024-10-15

**Authors:** Roman Vavrek

**Affiliations:** Department of Management, VŠB–Technical University of Ostrava, Ostrava, Czech Republic; University of Botswana, BOTSWANA

## Abstract

The performance evaluation of local self-government entities is very difficult, as their primary goal is not to make a profit, but to provide services to their residents that will contribute to an increase in their quality of life. In this context, it is necessary to evaluate their activity from the point of view of several available criteria, for which it is possible to find relevant and recognized sources. The presented research works with five criteria, identified by the Institute for Economic and Social Reforms, and aims to quantify the agreement of the results of the assessment of the financial health of territorial self-government entities in 2020 using the TOPSIS technique with a gradually decreasing number of criteria. For this purpose, a total of 26 combinations of criteria are created, with the number of 5, 4, 3 and 2 used criteria, the importance of which is determined based on their absolute variability using the standard deviation method. The results obtained in this way are interpreted using a wide range of mathematical and statistical methods including the Kolmogorov-Smirnov test, Levene test, Jaccard index and others. As a result, the multi-criteria evaluation of territorial self-government subjects (in our case, district cities) proved to be highly applicable. However, the result itself is largely determined by the structure and number of entry criteria. Based on the performed analyses, we can see that significant differences result from their reduction. Each such reduction has an impact on the overall results, but it is possible to find combinations that defy this conclusion.

## Introduction

Evaluating the performance of local self-government entities is very complex, which is a consequence of the fact that municipalities are not primarily supposed to make a profit, but rather to provide services to their residents that will contribute to an increase in their quality of life. The results of such municipal activities are largely intangible and often not easily observable or measurable [1, 2]. Measuring the success, or performance, of their activities is therefore especially demanding.

**Fig 1:**
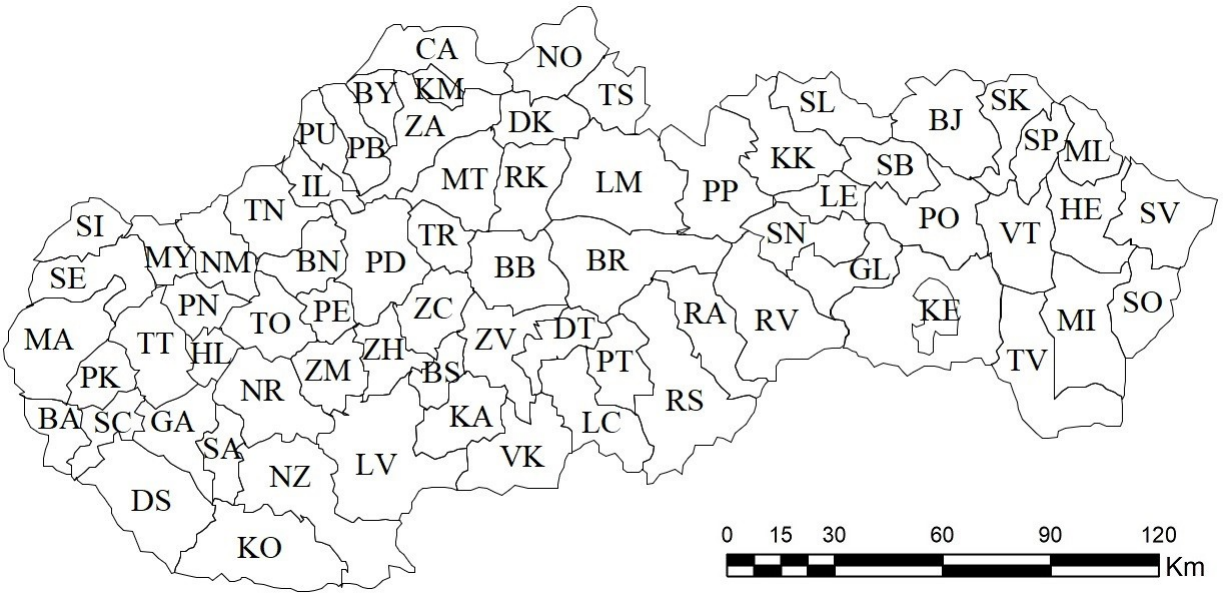
Territorial-administrative division of the Slovak Republic (districts).

Pursuant to Act no. 221/1996 Coll. on the Territorial and Administrative Organization of the Slovak Republic, its territory is divided into 8 regions and 79 districts; see the following map/figure (Fig 1).

Out of 79 districts, only 69 have district towns, the exception being 5 districts in the Bratislava self-governing region (Bratislava I district, Bratislava II district, Bratislava III district, Bratislava IV district, Bratislava V district) and 5 districts located in the Košice self-governing region (Košice I district, Košice II district, Košice III district, Košice IV district, Košice-okolie district). Apart from these 69 district towns, the Institute for Economic and Social Reforms also evaluates the financial health of the municipality of the capital Bratislava and Košice, i.e. the total number of evaluated subjects within the submitted research is 71.

Currently, in the conditions of municipalities, single-criteria methods are used sporadically. Among those used, we can include cost-benefit analysis (CBA), cost-effectiveness analysis (CEA), return on investment method (RI), payback method (PM), net present value method (NPV) and internal rate of return method (IRR).

Some authors [3] consider performance in the public sector and thus also in local government to be a multidimensional concept. Subjects moving in this environment should be evaluated based on several criteria. However, different applications are offered: in the form of a financial and property analysis assessment (FAMA) with 22 criteria [4]; in the form of a global competitiveness index with 12 pillars and a total of 102 criteria [5]; in the form environmental sustainability with 25 criteria [6]; in the form of fiscal rules and 13 criteria [7]; in the form of an assessment of financial autonomy of rural municipalities with 9 criteria [8]; and in the form of 10 European common indicators (ECI) [9]. In Slovak conditions, the issue was also addressed: in the evaluation of selected municipal services using 15 criteria [10]; in evaluating the use of the property of local governments using 6 criteria [11]; in evaluating the efficiency of municipal property used for business activity using 12 criteria [12]; in evaluating the management of municipalities using 8 criteria [13, 14]; and in the form of the financial health of municipalities and higher territorial unit (HTU) consisting of 5 indicators of financial stability [15].

All these approaches evaluate a selected group of subjects using a different number of criteria, starting with 5 and ending with a group with over 100 criteria. If the number of criteria is reduced, information is lost. What loss is acceptable? When working with a number of 100 criteria [5], a reduction of one criterion does not automatically imply a significant change in the overall results of the multi-criteria analysis. As the number of criteria decreases, their relative importance increases (1/n, when n = 100, 99, 98, …, 5, 4, 3, 2). For the purposes of this research, we have chosen the previously used financial health of municipalities and HTU [16] that works with 5 criteria, namely:

- Total debt,
- Debt service,
- Current account balance,
- Liabilities after the due date,
- Liabilities at least 60 days past due.

By reducing the number of criteria by one, 20% of the information is lost (1 in 5). The loss of one criterion increases the importance of the remaining criteria. Our interest here is to what extent the loss of one criterion affects the overall results. If the change is too large, it is not possible to reduce the criteria at all. If the change is too small, the use of the criterion is questionable. But what is the optimal number of criteria? Therefore, the research question of the present research is as follows:

Is there a minimum number of monitored criteria that does not result in a significant loss of information about the real state of the assessed entities?

The aim of the presented research is to quantify the agreement of the results of the assessment of the financial health of the entities of the territorial self-government in 2020 using the TOPSIS technique with a gradually decreasing number of criteria.

To fulfil the set goal, the presented article is divided into sections as follows. The first section is the Introduction. The second section is devoted to an introduction of the TOPSIS technique as the main methodological tool of the presented research. The third section describes a set of five evaluated criteria using selected moment characteristics. Its content also includes the identification of the structure of 26 combinations of criteria with a decreasing number, which are subsequently the subject of further evaluation. In a separate sub-section, attention is also directed to determining the weight of the criteria, which directly affects the result of the multi-criteria analysis. The content of this section also includes the identification of the research sample and the mathematical and statistical methods used to evaluate the obtained results. These are described and interpreted within the fourth section, which is processed using 5, 4, 3 and 2 entry criteria. Summarizing the knowledge gained is the content of the fifth and last section, which is the Conclusion.

## Literature review

Need of comparing MCDM methods and the importance of selection for subsequent analysis for the first time was first explored in 1968 [17]. It is possible to find a number of studies that addressing this issue [18, 19]. One of them proposed a methodological approach to selecting appropriate MCDM method for a particular decision-making situation [20]. Subsequently, they were other studies comparing the appropriateness of using different methods have been published [21, 22]. Most of these approaches are based on restrictive assumptions regarding the structure of preferences of the decision maker or are developed to address problems of special structure, e.g., many (qualitative) criteria and few alternatives [23, 24] or few quantitative criteria and many alternatives [25, 26]. In general, it is very difficult to decide which technique is the most appropriate [27, 28] and depends on the specific conditions of the decision project.

The TOPSIS technique (Technique for Order of Preference by Similarity to Ideal Solution) is one of the basic methods of multi-criteria decision-making, and its primary use is in solving different types of decision-making problems. This method is one of the most widely used [14], with the SAW, AHP, or VIKOR methods as possible alternatives. A theoretical comparison is already processed [29]. Results of practical applications [30] show that TOPSIS produces rankings which are extremely similar to the ones resulting from SAW. Compared to the VIKOR method, TOPSIS method introduces two “reference” points, but it does not consider the relative importance of the distances from these points [31]. In the comparison between the VIKOR and TOPSIS methods, 84.1% (data in uniform distribution) and 82.3% (data in normal distribution) of the ranking results were the same, and in the comparison between the proposed method and VIKOR, 83.7% (data in uniform distribution) and 83.7% (data in normal distribution) of the ranking results were the same [32]. The TOPSIS approach is less predictive than the AHP model [33]. Table 1 offers a simple comparison with other MCDM methods.

**Table 1:** Comparison of the selected multi-criteria methods.

Attributes	TOPSIS	SAW	AHP	VIKOR
International practice for addressing economic objectives	Applicable	Applicable	Not applicable	Applicable
Measurement dimensions for different criteria	Available	Available	Available	Not available
Complexity of the method	Complex	Simple	Average	Complex
Objective structure	Linear, Non-linear, Vector	Linear	Hierarchic	Linear
Assessment of qualitative criteria	Available	Available	Available	Available
Assessment of quantitative criteria	Available	Available	Not available	Available
Method for identification the best alternative	Closeness to the PIS	Weighted	T. Saaty method	Closeness to the PIS
Labour costs	High	Low	Average	Hight

An overview of its applications was captured by many authors [34, 35], who noted an annually increasing number of articles in which the use of the TOPSIS technique could be found. The choice of the TOPSIS technique for the purposes of our research was based on its previous successful use in solving decision-making problems of a similar nature. You can find its applications in environmental science, where this technique was used for the evaluation of electricity policy and progress in renewable energy technologies usage [36–38]. It was used also in transport sector for energy-environment efficiency of European transport sectors evaluation and efficiency of transport companies in the Czech Republic [39, 40]. The comparison of local government units [41] and culture units [42] were other examples of successful application of this approach. We are able to find many other areas of its successful applications that support our decision to select this method for our research, see [43–49].

**Fig 2:**
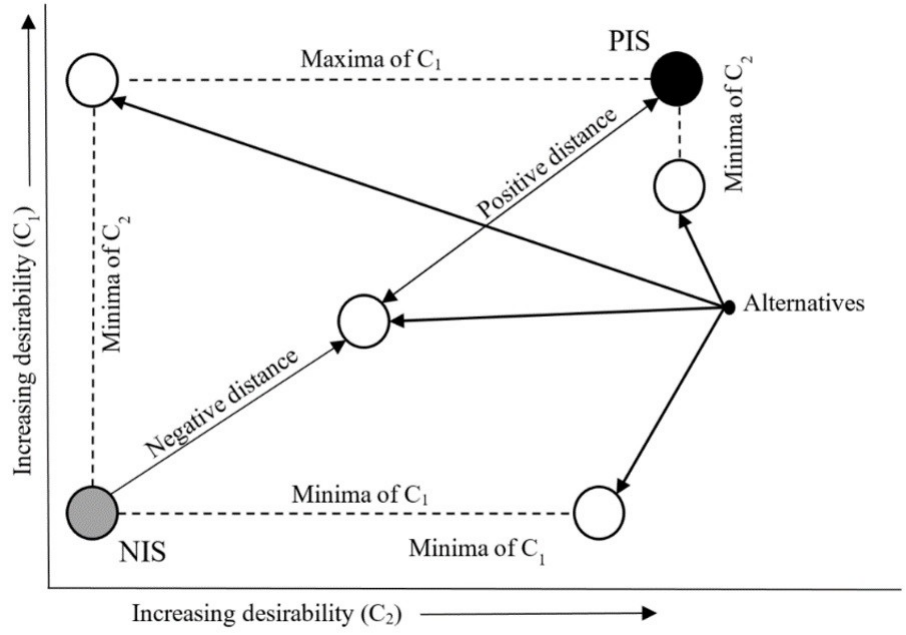
The principle of operation of the TOPSIS technique.

The principle of the TOPSIS technique could be illustrated using the above figure [34], see Fig 2. Each white ball represents one specific alternative, i.e. one evaluated subject. The grey ball represents the Negative Ideal Solution (NIS) alternative, i.e. a real or hypothetical alternative (subject) with the worst values of the individual criteria. The black ball represents the Positive Ideal Solution (PIS) alternative, i.e. a real or hypothetical alternative (subject) with the best values of the individual criteria. The alternative (one of the white balls) that is farthest from the grey ball (NIS) and closest to the black ball (PIS) is the best rated.

This method is a suitable decision-making tool based on incomplete data [50]. The range of selected data is not decisive for its use, i.e. it is possible to use data of any range [51, 52]. The strengths include straightforwardness and simplicity of calculation [53, 54], the ability to work with all types of criteria [52], and complexity [55]. The main disadvantage of the TOPSIS method is considered to be the absence of the possibility of assigning weights to the monitored criteria and the absence of consistent control by the decision-maker [51]. For this reason, this method is dependent on the process of finding the relative importance of different criteria, which is also the area of interest of the presented research. Many authors [56–58] deal with the calculations of the TOPSIS technique itself in more detail.

In general, it can be stated that the result of the application of the multi-criteria decision-making (MCDM) method is directly determined by the person making the decision (by the decision-maker). The process of determining the importance of the evaluated criteria is very important [59], as weights affect the final arrangement, and an improper determination of the weights can lead to a change in the arrangement, including what approach or method is chosen for determining the importance of individual criteria. One classification of these methods identifies two groups of methods [60]. Other one works with three groups [58], while the third one [61] uses four groups of weighting approaches that represent an extension of the previous classifications. The groups in question are as follows:

- subjective methods,
- expert methods,
- objective methods,
- integrated methods.

Subjective methods reflect the personality of the decision-maker and his/her individual preferences (the weight of the indicator is determined on the basis of subjective opinion). Expert evaluation is carried out by a smaller number of experts in the given field, while an application from the past is offered [62–64]. The use of the opinion of an expert group using a pairwise comparison of criteria (e.g. using Fuller’s triangle) can be found in several researches [65–67]. The third group, i.e. a group of objective methods, assigns weight to individual criteria based on a predetermined mathematical model unique to each method. Therefore, the decision-maker does not have a direct influence on determining the importance of the criteria. She/He chooses, according to personal preference, the attributes of the data used, e.g. according to the variability or relationships between criteria. In this group it is possible to find methods such as the mean weight method [68–70], standard deviation method [41, 71], Mahalanobis-Taguchi system method [72], λ bi-capacity model [73], coefficient of variance method [74] and others [75, 76]. The last group presents integrated methods, which represent a combination of the already described approaches. Other approaches are presented in actual research applied in different areas, e.g., AHP according to experts’ views [77], a linear regression model [78], KEMIRA-M procedure [79], MOWSCER method [80], N-CRITIC method [81], BWM method [82], etc.

## Materials and methods

The financial health of a district city calculated by the third side [15] provides information about sustainable management and whether or not municipal management is causing problems for the respective city. This rating is “normed”, representing a single number between 0 (worst) and 6 (best). More detailed information on the calculation of individual indicators can be found on the website.

Our intention is not to challenge this calculation–quite the contrary. In the previous section, different approaches to the evaluation of the management of the basic entities of territorial self-government were presented, which worked with a different number of entry criteria. Compilation of a relevant set of evaluated criteria is a significant factor influencing the final evaluation in any evaluation process. Our research works with the entry criteria of this financial health [13], which is calculated as a weighted average of the scores achieved by each of its five components:

- K1 ‐ Total debt,
- K2 ‐ Debt service,
- K3 ‐ Current account balance,
- K4 ‐ Liabilities after the due date,
- K5 ‐ Liabilities at least 60 days past due.

All criteria represent a relative variable expressed in percentages, i.e. from this point of view, it is a homogeneous group, the parameters of which can then be easily compared with each other.

Our methodology can be described using the following steps (phases):

- Literature review I. ‐ based on the comprehensive literature review, the TOPSIS technique is selected as a main research tool,
- Literature review II. ‐ based on the comprehensive literature review, the SD method is selected as tool for determining the importance of entry criteria (in association with the TOPSIS technique),
- Data preparation ‐ 5 criteria are selected and processed for the needs of use using the above methods,
- Analysis realization ‐ 26 separate analysis is realized to achieve the aim of this research (A, B1-B5, C1-C10, D1-D10),
- Conclusion formulation ‐ based on the results achieved, the limitations and potential further research topics are formulated.

### Comparison of the evaluated criteria in the light of moment characteristics

The use of criteria in a relative form (as a percentage) enables their simple comparison without the need for their normalization or other modification. The moment characteristics described above reflect the attributes of the individual criteria, which can be compared through the following pair of figures.

**Fig 3:**
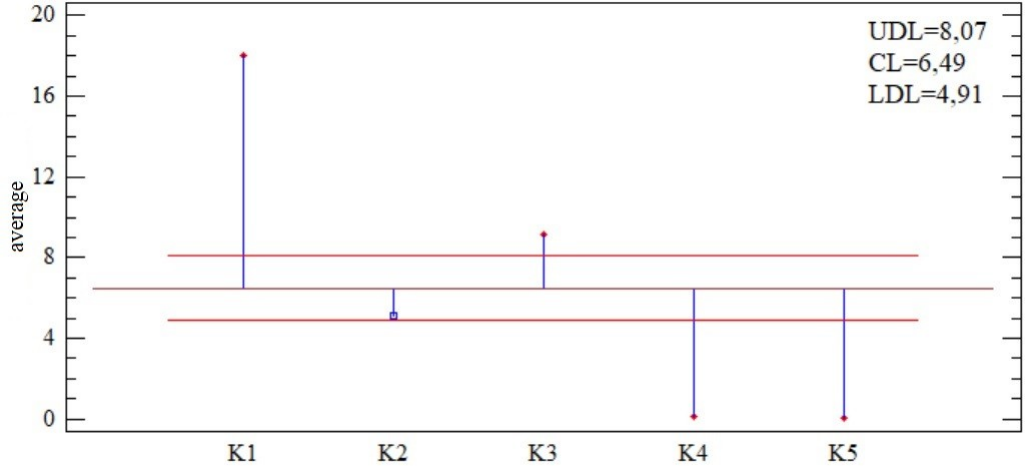
ANOM analysis of evaluated criteria K1–K5.

The first one (Fig 3) offers the results of the ANOM analysis (analysis of mean), i.e. it compares the average of individual criteria with the common average for all criteria. From this point of view, the K1 criterion is dominant, as the arithmetic mean is at the level of 18%. We observe the opposite trend in the case of criteria K4 and K5, for which the average oscillates around the zero value; this is due to the high number of district towns with a zero value for the monitored criterion.

The mean value shown on the previous figure also indicates the distribution of individual observations (district towns) across the variation range of that criterion. The most even use of the available variation range is also offered by the criterion with the highest mean value, i.e. total debt (K1), as mentioned above. In the case of criteria K4 and K5, we observe the most subjects with the same value (zero), which is reflected in the quantile plot in the form of a significant vertical curve for both criteria.

**Fig 4:**
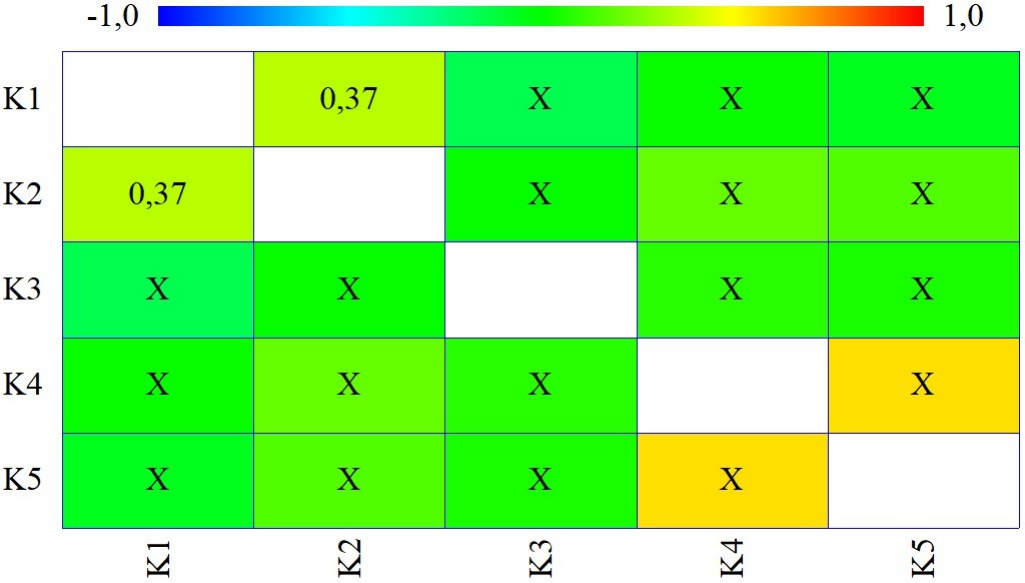
Results of correlation analysis using Kendall’s rank correlation coefficient.

The individual criteria have significantly different attributes, which indicates their added value and justification for subsequent use within the intended multi-criteria analysis, as they are unique in their structure and do not repeat each other. This fact is also documented by the following figure (Fig 4), with contains the results of the rank correlation analysis. Only in one case is there a positive rank correlation between the pair of criteria K1 and K2, but we can call it small.

### Combinations of entry criteria as a structure of our own research

The primary result of the performed analysis is given by the TOPSIS technique using all 5 criteria, which represents the “benchmark” of individual analyses. Thus, the set of criteria is given, and as such represents combination A (see Table 2).

**Table 2:** A set of 5 criteria for the needs of multi-criteria evaluation–combination A.

	combination	criteria included in the analysis				
**5 criteria**	A	K1	K2	K3	K4	K5

The results obtained with a smaller number of criteria are compared with these results in the following structure. In the first part of the analysis, one criterion is removed from these 5 criteria each time, i.e. multicriteria analysis is processed using 4 criteria a total of 5 times (combinations B1 –B5). The structure of each combination is captured in the following table (Table 3).

**Table 3:** A set of 4 criteria for the needs of multi-criteria evaluation ‐ combinations B1– B5.

	combination	criteria included in the analysis				
**4 criteria**	B1	K1	K2	K3	K4	
	B2	K1	K2	K3		K5
	B3	K1	K2		K4	K5
	B4	K1		K3	K4	K5
	B5		K2	K3	K4	K5

In the second part, the results are obtained using three monitored criteria, i.e. in each case, 2 of the 5 criteria are removed. A total of 10 combinations are thus created, each of which represents a separate calculation, or analysis (see Table 4).

**Table 4:** A set of 3 criteria for the needs of multi-criteria evaluation ‐ combinations C1–C10.

	combination	criteria included in the analysis				
**3 criteria**	C1	K1	K2	K3		
	C2	K1	K2			K5
	C3	K1			K4	K5
	C4			K3	K4	K5
	C5	K1	K2		K4	
	C6	K1		K3		K5
	C7		K2		K4	K5
	C8	K1		K3	K4	
	C9		K2	K3		K5
	C10		K2	K3	K4	

In the third and last step, the TOPSIS technique is calculated using only a pair of criteria, while the other 3 criteria are not considered. By choosing 2 criteria out of 5, we get 10 combinations, which represent further processed combinations C1 –C10 within the presented research (see Table 5).

**Table 5:** A set of 2 criteria for the needs of multi-criteria evaluation ‐ combinations D1–D10.

	combination	criteria included in the analysis				
**2 criteria**	D1	K1	K2			
	D2	K1		K3		
	D3	K1			K4	
	D4	K1				K5
	D5		K2	K3		
	D6		K2		K4	
	D7		K2			K5
	D8			K3	K4	
	D9			K3		K5
	D10				K4	K5

In total, each of the performed analyses consists of the performance of 26 calculations, taking into account the above-mentioned structure of entry criteria. The obtained results are compared with each other using a wide set of mathematical and statistical methods described in other parts of this section.

### The importance of criteria in the context of their decreasing number

The presented research works with an approach to determining the importance of evaluated criteria, which represents a representative of objective methods for determining the importance of criteria. This approach works with the absolute expression of the variability of the evaluated criterion, while the obtained results are therefore influenced by the values of the moment characteristics of the level and position. Its previous successful usages can be found.

The group of objective methods consists of methods that work with the moment characteristics of the criteria in order to determine their importance. The simplest way is to use the variability of the criteria, either in absolute or relative terms. All other approaches require additional calculations, increasing the complexity on the part of the decision maker [77–83].

The importance of entry criteria is determined based on variability in absolute as the simplest approach for weight of criteria determination. The importance of the evaluated criteria is determined based on the standard deviation method (i.e. standard deviation method, hereinafter referred to as “SD”).

$$w_{j}=\frac{SD_{j}}{\sum_{j=1}^{n}SD_{j}}$$ (1)

where: SD_j_ = standard deviation of j(th) criterion

n = number of criteria

Using this approach and the moment characteristics described in the previous section, the importance of the criteria in individual combinations is calculated.

**Fig 5:**
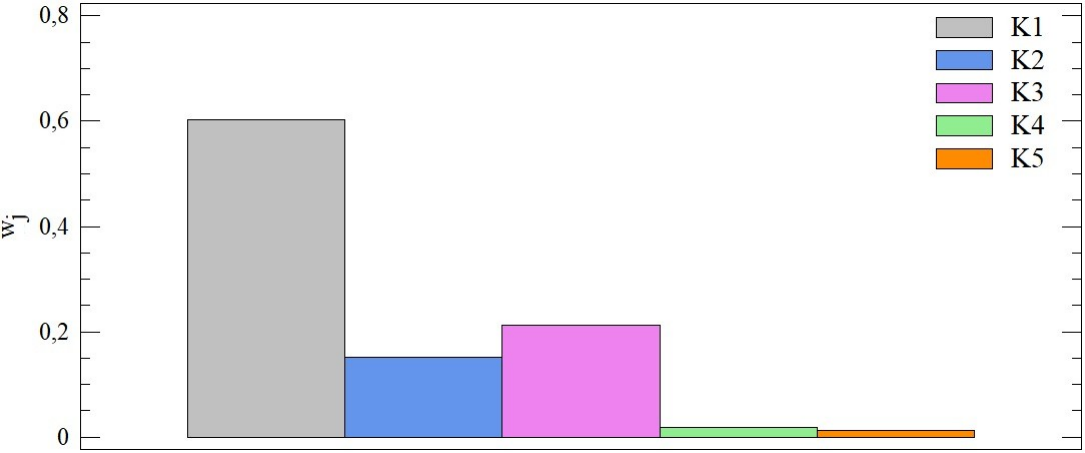
The importance of entry criteria from the point of view of the SD method ‐ combination A.

The principle of this method used for determining the importance of criteria is based on measuring their absolute variability through one of the momentary characteristics of variability, namely the standard deviation. Within combination A, district towns are evaluated using 5 criteria, where this approach gives the highest importance to the first of these criteria, i.e. Total debt (w_1_ = 0.602). This is followed by a pair of criteria with importance at the levels of 15.21% and 21.32%. From this point of view, the significance of the remaining two criteria is minimal and, in both cases, does not exceed 2% (see Fig 5).

**Fig 6:**
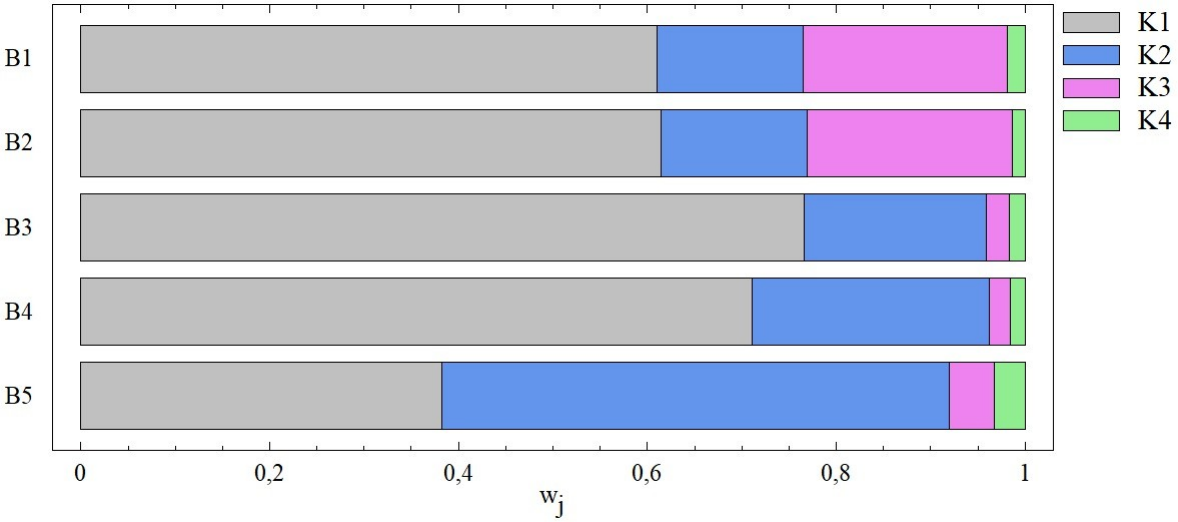
The importance of entry criteria from the point of view of the SD method ‐ combinations B1 –B5.

In the next step, we work with 4 criteria (combinations B1-B10), where the first criterion is always dominant (criterion first in order, not K1). Its importance, apart from combination B5, exceeds 60%, while in combinations B3 and B4 its dominance is even higher (w_1_ = 0.765 and w_1_ = 0.710, respectively). In all these cases, it is one and the same criterion, namely Total debt (K1), which mostly affects the results of the multi-criteria evaluation using the TOPSIS technique. We see a different situation in the last combination, B5, where the dominance of the first criterion decreases; this is caused precisely by the absence of the K1 criterion. However, the importance of the last criterion is always minimal, which is also illustrated by the following figure (Fig 6).

**Fig 7:**
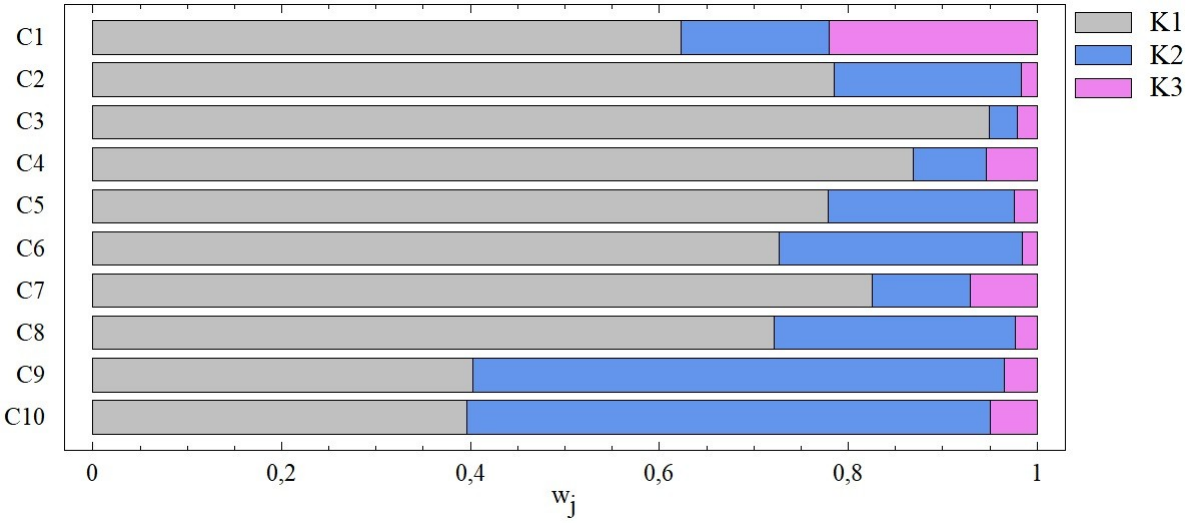
The importance of entry criteria from the point of view of the SD method ‐ combinations C1 –C10.

Combinations marked C consider the three entry criteria, see Fig 7. In most cases (C1-C8), we observe the dominance of the first criterion, which is one of the criteria K1, K2, K3. The combination C3 is interesting, in which the dominance of the first criterion is most pronounced when the weight of the other 2 criteria does not exceed 3%. With combinations C9 and C10, it is possible to talk about the balance of the two most important criteria. The small influence of the third criterion on the overall results of the TOPSIS technique persists regardless of the degree of dominance of the first criterion.

**Fig 8:**
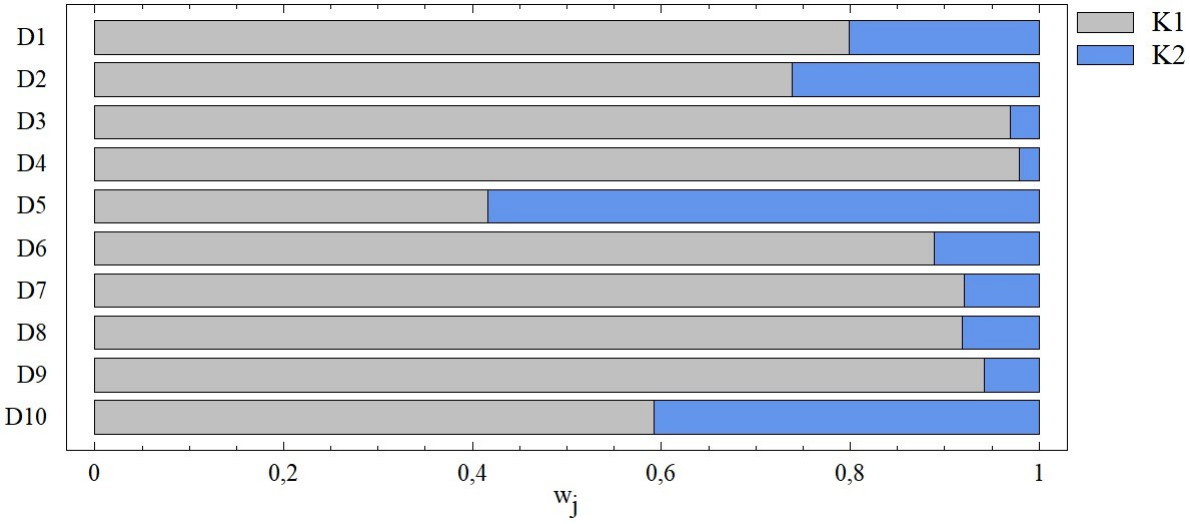
The importance of entry criteria from the point of view of the SD method ‐ combinations D1 –D10.

Combinations D1 –D10 are mostly characterized by the dominance of one of the two evaluated criteria, see Fig 8. In five cases (D3, D4, D7, D8, D9), the importance of the second criterion does not even reach the level of 10%, i.e. the overall result is primarily influenced by the more significant of the given pair of criteria. Only in the case of the D10 combination, including criteria K4 (Liabilities past due to income) and K5 (Liabilities at least 60 days past due), can we call these criteria relatively equivalent.

From the point of view of individual combinations, we observe significant differences in the importance of the criterion determined by the SD method. The dominance of criterion K1, which was the criterion with the highest importance values in 15 out of 26 combinations, is interesting. On the other hand, it was not the least important criterion in any case, regardless of whether 5, 4, 3 or 2 criteria were evaluated. We observe the opposite situation in the case of Liabilities at least 60 days past due (K5), which is the exact opposite of the above-mentioned criterion. This criterion is not the most important in any combination, but in 15 cases it was the criterion with the lowest importance (i.e. in all combinations of which it is a part), see Table 6.

**Table 6:** Identification of the most important and least important criterion using the SD method ‐ all combinations.

criterion	the most important	the least important
K1	15 (57,69%)	0 (0,00%)
K2	3 (11,53%)	3 (11,53%)
K3	7 (26,92%)	1 (3,84)
K4	1 (3,84%)	7 (26,92%)
K5	0 (0,00%)	15 (57,69%)

### Description of the apparatus of the mathematical and statistical methods used

A wide range of mathematical and statistical methods is used for processing the obtained results, a description of which can be found in several sources [84–88]. The only differences are the designations used in individual publications.

The basic prerequisite for the selection of appropriate methods in any statistical investigation is to identify whether or not the investigated data come from a normal distribution. This assumption subsequently serves as a starting point when choosing from parametric or non-parametric statistical methods. The normality of the distribution of the TOPSIS technique, resulting from the use of individual combinations (A, B1-B5, C1-C10, D1-D10), is monitored by the Shapiro-Wilk test [89].

The starting point and thus the tested sets for statistical processing are the results obtained by applying the TOPSIS technique. The comparison of the order obtained in this way is carried out by means of Kendall’s rank [62] correlation coefficient (hereinafter referred to as “Kendall’s coefficient”). This coefficient represents a non-parametric alternative to the frequently used Pearson’s correlation coefficient [90] (hereinafter referred to as “Pearson’s coefficient”), which also tracks the correlation between two numerical variables.

The strength of the Kendall coefficient is lower, but it is useful in the event of extreme values that significantly affect the value of the regression coefficients and thus the estimation of the residuals. The Kendall coefficient is automatically used in the analysis in the case of monitoring the correlation of two numerical variables when the condition of using the Pearson coefficient is not met, i.e. normality of both observed variables (tested by the aforementioned Shapiro-Wilk test).

Another subject of testing is the agreement of the results of the TOPSIS technique using five criteria (combination A) with other results (combinations B1-B5, C1-C10, D1-D10), which is established by testing the agreement of distribution functions using the Kolmogorov-Smirnov test [91]. This test has two variants; for the purposes of our own research, the second one is used, i.e. we compare two distribution functions and verify their agreement.

The agreement of the standard deviation of the two indicated above, i.e. homoscedasticity, is verified through the Levene test [92]. The agreement of the obtained results is also monitored at the level of the ranking of individual subjects, whose agreement is quantified by means of the Jaccard index.

When estimating the spatial association, first, the spatial relations between territorial units/areas must be defined. They describe their spatial weights calculated from point or polygon data, although the method of calculation differs. Spatial weight is a basic element of spatial statistics for measuring spatial ties. Spatial weight matrices represent the strength of potential interaction between individual places, or between spatial units [93]. Rook-type weights are used, so only areas adjacent to the investigated area are taken into account, which is also reflected in the neighbourhood matrix. After identifying the spatial weights, Moran’s local coefficient [94] is used, which determines whether there is spatial autocorrelation for a given set of regions, or territory.

The one-sided relationship between the dependent (results of the TOPSIS technique) and the independent variable (number of inhabitants) is verified on the basis of a simple regression model, whose explanatory power is quantified using the coefficient of determination [95]. The obtained results are described using selected moment characteristics [96].

All analyses are processed using the software MS Office Excel, Statistica and Statgraphics.

## Results and discussion

**Fig 9:**
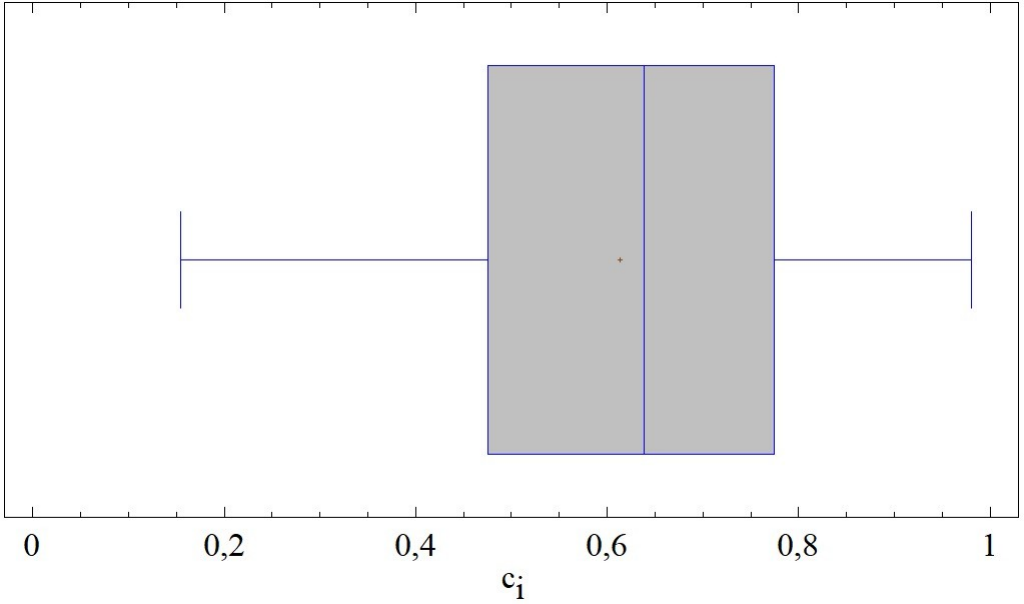
Results of SD-TOPSIS application ‐ combination A.

At the determined level of significance, the normality of the results was confirmed (S-W = 0.965; p = 0.05), while the results themselves are slightly negatively skewed compared to the Gaussian curve, with a smaller concentration around the mean value (β = -0.498). The interquartile range at the level of 0.299 c_i_ represents more than 36% of the total variation range, which makes it possible to expect significant differences between the evaluated district towns. No extreme or outlier value appears in the results, which is precisely due to the relationship of the above-mentioned ranges (see Fig 9).

From the point of view of the distribution of results in space, we note their randomness, or spatial uncorrelation (M = 0.0004). The best-rated district towns are located primarily in Central Slovakia, with the absolute best being Tvrdošín (0.980 c_i_), followed by Poltár (0.928 c_i_) and Nový Mesto nad Váhom (0.910 c_i_). Half of the 10 worst-rated district towns can be found in Eastern Slovakia; the rest are scattered across the entire territory of Slovakia. Table 7 contains its statistical description.

**Table 7:** Description of results of SD-TOPSIS application (combination A) using selected moment characteristics.

characteristics	value
Average	0.613744
Median	0.63859
Minimum	0.153953
Maximum	0.980158
Range	0.826205
Lower quartile	0.475734
Upper quartile	0.774962
Skewness	-0.468656
Kurtosis	-0.498931

The relationship between the obtained results of the multi-criteria evaluation and the number of inhabitants of the district towns can be quantified through a simple regression model (using OLS method) as follows:

$${\text{SD-TOPSIS}}_{\text{A}}=\left(0,0766953*\text{In}(\text{NI}){)}^{2}\right.$$ (2)

where: NI = number of inhabitants

The degree of variability of the dependent variable in such a model is significant (R^2^ = 95.69%), i.e. the number of inhabitants has a positive effect on the overall results of the SD-TOPSIS technique when including five entry criteria (combination A).

### Evaluation of district towns using four entry criteria (combinations B1-B5)

**Fig 10:**
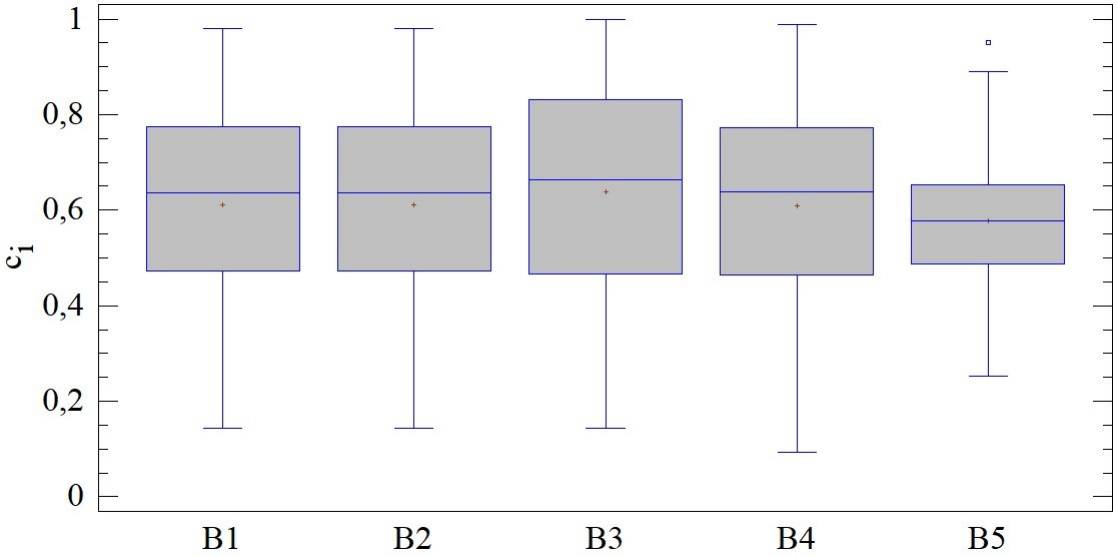
Results of SD-TOPSIS application ‐ combinations B1-B5.

The results of the SD-TOPSIS technique when including 4 out of 5 input criteria (combinations B1-B5) show similar properties regardless of the omitted criterion, as in combination A. In 4 out of 5 cases the results are slightly negatively skewed, which corresponds to the position of the mean and the median. The highest average value is achieved by the combination B3 (0.638 c_i_), while the lowest is achieved by the combination B5 (0.577 c_i_); however, this is due to the overall lower results of the second mentioned combination. We observe significant variability measured by either the coefficient of variation or the standard deviation, which is given by a lower concentration of results around the mean value and a considerable interquartile range. The rejection of the assumption of homoscedasticity of the results can be attributed to the combination B5, which is significantly different from the results of all other combinations (LE = 6.478; p < 0.01). For more details, see Fig 10.

The high degree of similarity in the structure of the results of B1-B4 with the results of combination A can be captured using correlation analysis, specifically the Kendall coefficient (see the following table), which can be used to classify it as almost perfect (min. r_K_ = 0.933). A district town with a high rating using 5 criteria also achieves a high rating using 4 criteria. In the case of combination B5, this correlation is also statistically significant, but its strength is significantly lower.

The correlation analysis is supplemented by a comparison of distribution functions through the Kolmogorov-Smirnov test. As can be read from the following table, in the case of 3 combinations (B1, B2, B4) we can talk about a perfect match of the distribution of the obtained results. It can initially indicate the substitutability of the combinations. Differences in results can be assumed for the combination B5, for which a statistically significant difference was confirmed.

**Fig 11:**
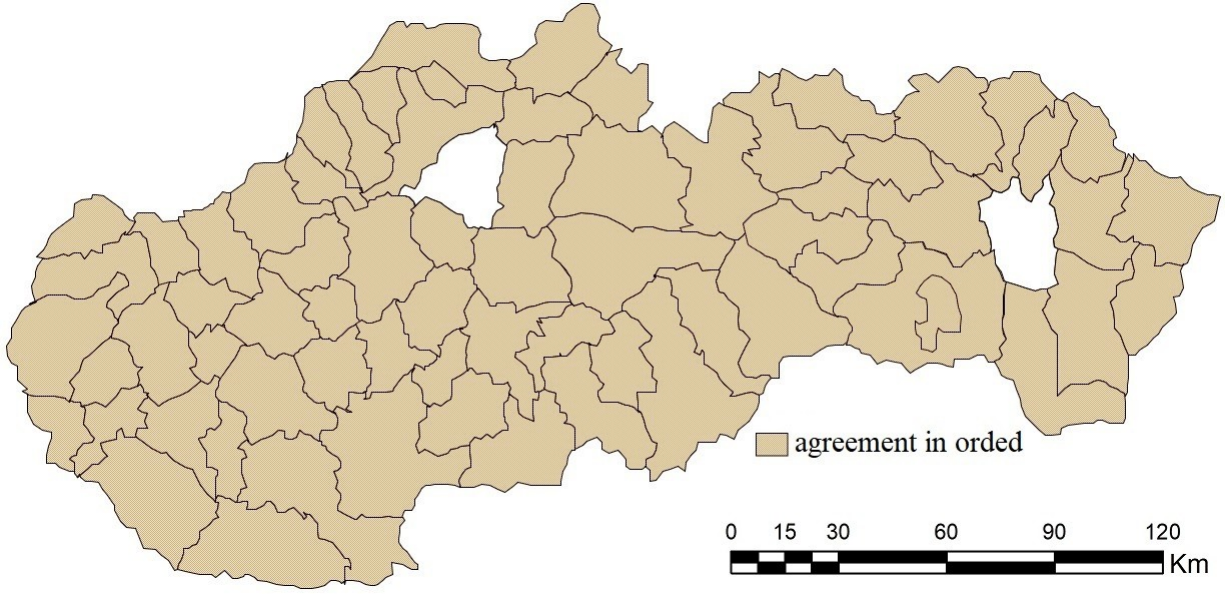
Agreement in the order of district towns in the results of the SD-TOPSIS application in space ‐ combination A vs. B1.

Taking a closer look at the results of specific district towns, it is possible to find significant differences across the evaluated combinations. The Jaccard index points to an agreement in the order of district results exceeding 90% in the case of combination B1 (97.18%) and combination B2 (94.36%); see Fig 11. In the other 3 combinations, this agreement is significantly lower, while in combination B5 it is at the level of 2.81%. The order is therefore significantly heterogeneous within the individual combinations, which enhances the importance of the selection of entry criteria when evaluating their importance using the standard deviation.

**Fig 12:**
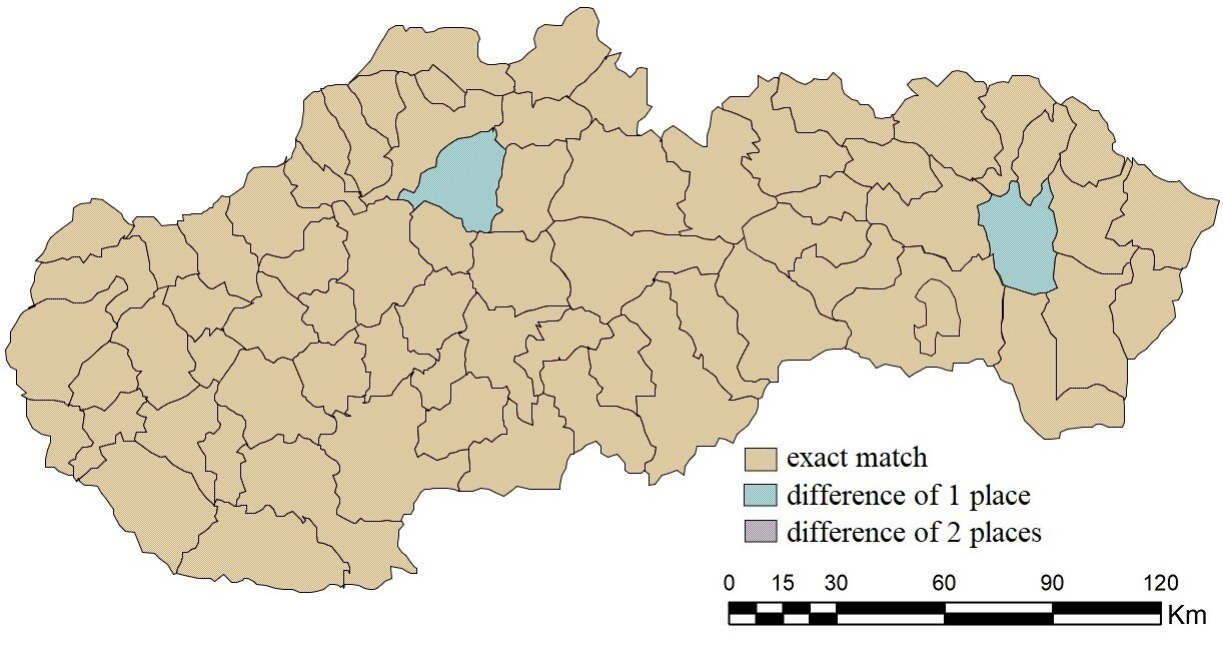
Agreement with tolerance in the order of district towns in the results of SD-TOPSIS application in space ‐ combination A vs. B1.

Due to the small differences in the results, the agreement in the order needs to be perceived more loosely, i.e. via accepting deviations. In the case of acceptance of the difference of one place in the overall ranking, the similarity of the results increases with the B1 combination on 100%, since Vranov nad Topľou and Martin differed by only 1 place. We also observe a strong agreement in the case of the combinations B2 (97.18%) and B4 (69.01%). If we accept a difference of 2 places in the overall order, the agreement in the case of combination B2 would also be 100%, while in combination B4 we get to 87.32%. The combination of B5 with 12.67% turns out to be completely inappropriate. For detailed results, see Fig 12.

When evaluating the results of the SD-TOPSIS technique in the context of the size of the evaluated subject, the comparison is carried out by a regressor and a coefficient of determination; see the following table. The high informative value of individual regression models remains, as well as the positive influence of the number of inhabitants on the evaluation results. Across the individual combinations, we do not record a significant difference in this influence, which is also documented by the stable level of the regressor.

### Evaluation of district towns using 3 entry criteria (combinations C1-C10)

**Fig 13:**
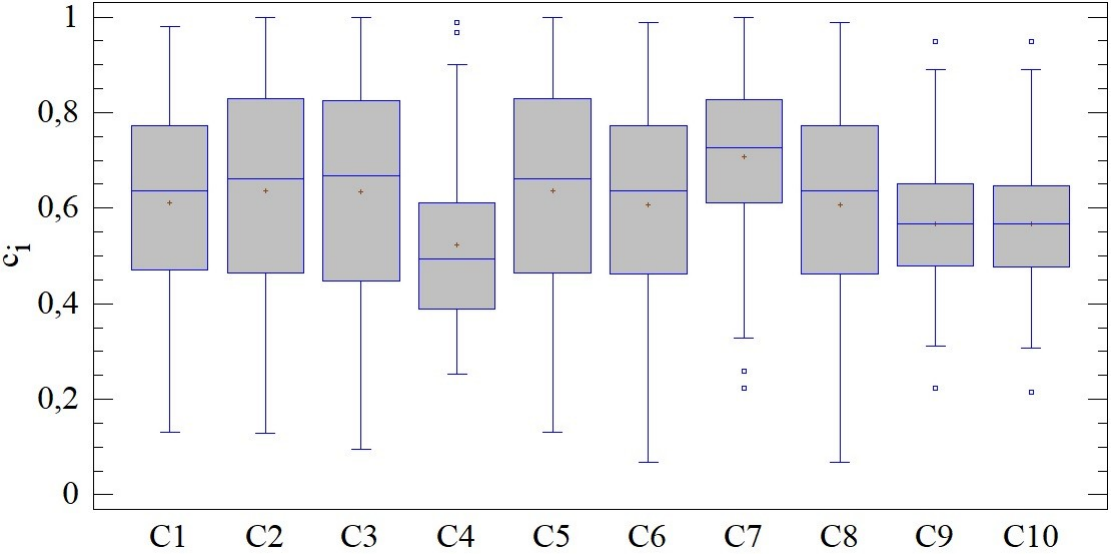
Results of SD-TOPSIS application ‐ combinations C1-C10.

When evaluating the results of the SD-TOPSIS technique including 3 of the 5 entry criteria (combinations C1-C10), we observe differences in the position of the mean values, variability or shape (see Fig 13). The variation ranges from 0.726 c_i_ (C9) to 0.922 ci (C6). In 4 out of 10 combinations, it does not include the results of all district cities (identified outliers). In an absolute sense, the best results are achieved with the C7 combination, where we also observe their slightly negative skewness. The C2 combination has the most significant differences between the evaluated subjects; however, this does not mark the individual results as outliers. These differences lead to the rejection of the assumption of homoscedasticity of the results, which cannot be confirmed (LE = 6.765; p < 0.01) due to significant differences in the total number of 20 pairs of combinations, e.g. C1-C9, C3-C4, C6-C10 and others.

The relationship between the results of the initial combination A and combinations using 3 criteria is captured using the Kendall coefficient, i.e. using rank correlation analysis. From this point of view, the evaluated combinations can be divided into two groups. The first one, which includes combinations C1, C2, C3, C5, C6 and C8, is characterized by a very strong and almost perfect linear connection of the results of the SD-TOPSIS technique. The largest significant correlation is measured in the pair A-C1, while the smallest has a value of 0.917 (A-C3). The second group, including combinations C4, C7, C9 and C10, has a statistically significant rank correlation, but with a much lower strength.

The agreement in the structure of the obtained results of the SD-TOPSIS technique is verified by comparing the distribution functions offered by the following table. The agreement of the distribution functions can be found in 6 out of 10 pairs, specifically when comparing the results of combination A with the results of combinations C1, C2, C3, C5, C6 and C8. In other cases, the differences are statistically significant, which points to differences in the results of individual subjects with these combinations. In the group of 10 combinations with 3 entry criteria, it is possible to find those that largely copy the distribution of the results of combination A (with confirmed agreement of distribution functions). In other cases, the distribution is different; the most significant differences are observed in the case of the C4 combination.

**Fig 14:**
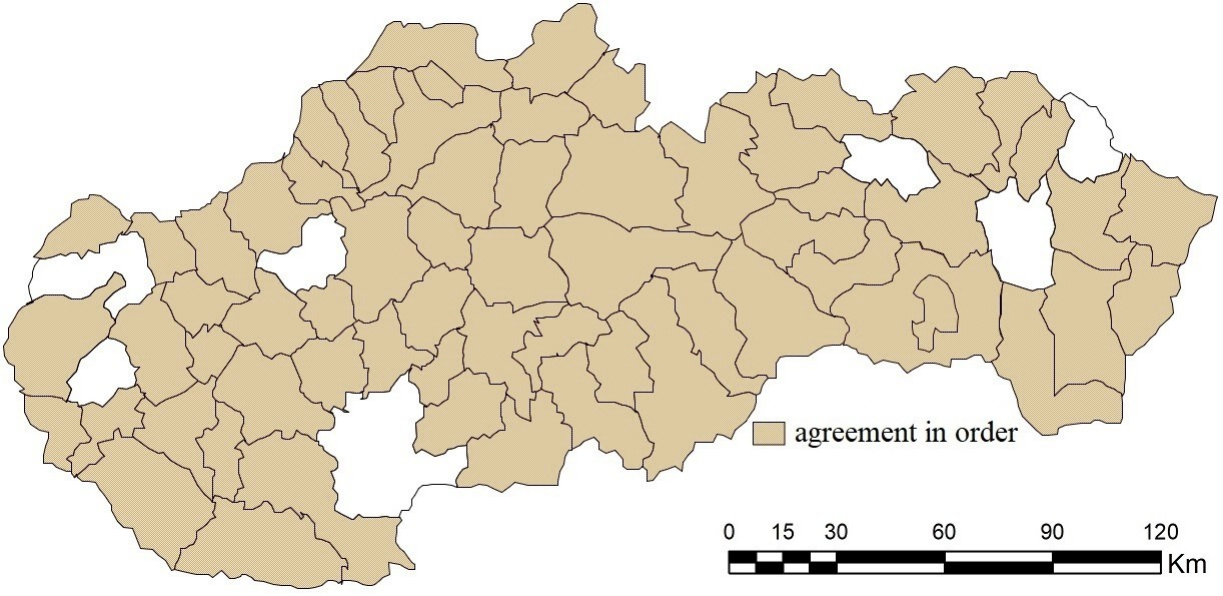
Agreement in the order of district towns in the results of the SD-TOPSIS application in space ‐ combination A vs. C1.

The relevant differences in distribution functions also portend a different degree of agreement in the order of district towns, which is quantified for the needs of the presented research by the Jaccard index (see Fig 14). Its highest values are observed in the case of combination C1 (90.10%), which is closely followed by combinations with an agreement oscillating around 30%, namely C6 (29.57%) and C8 (30.98%). In other cases, the differences in the order are striking; the most significant ones are observed in the combinations C7, C9 and C10 at the level of 2.81%.

**Fig 15:**
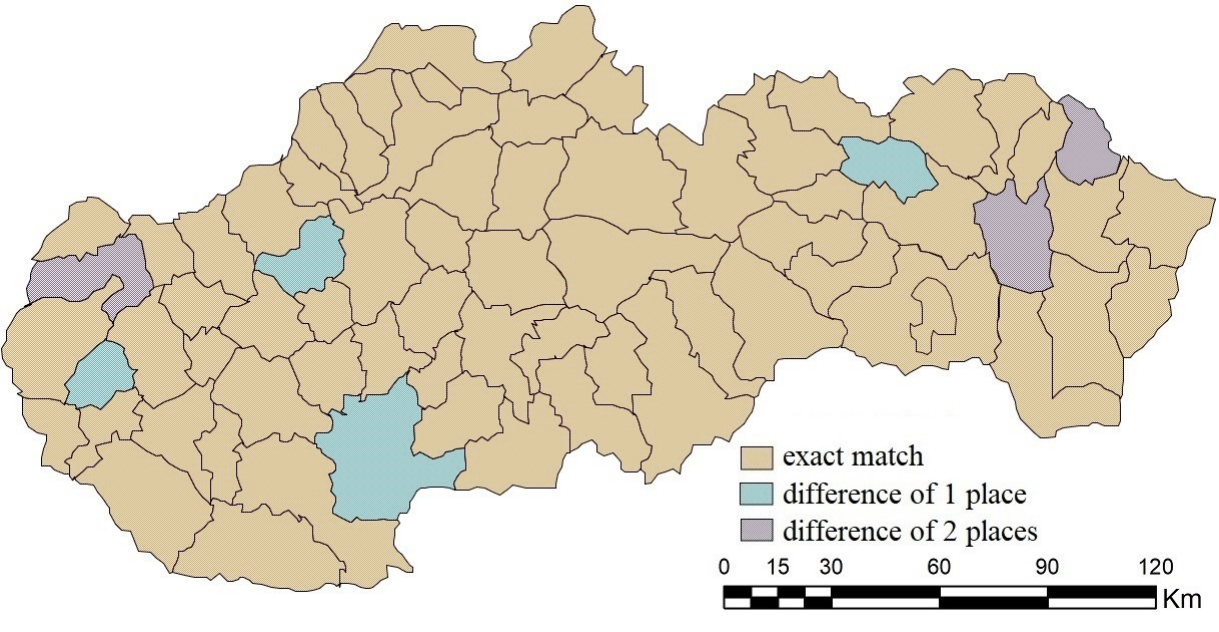
Agreement with tolerance in the order of district towns in the results of SD-TOPSIS application in space ‐ combination A vs. C1.

By accepting the difference of one place in the order of results, the similarity of the order of district towns in individual pairs increases, and the combination C1 again has the highest one (95.77%). Bánovce nad Bebravou, Levice, Pezinok and Sabinov were added to the 64 subjects with an exact matching order. The highest increases are recorded for combinations C6 and C8 (by 38.02 p.p.), and C2 and C5 (by 36.61 p.p.), in the case of which we can see an agreement exceeding 50%. With a tolerance of a difference of 2 places, combination C1 completely copies the ranking of combination A, see Fig 15. For the needs of further research, it is also necessary to consider combinations C6 and C8, with a match exceeding 85%.

The results of the SD-TOPSIS technique can also be evaluated in the context of the size of the evaluated subject, while the comparison itself is carried out through the regressor and the coefficient of determination, i.e. parameters of a simple regression model. The high informative value of the individual models remains, as well as the positive influence of the number of inhabitants on the evaluation results. Across the individual combinations, we do not register a significant difference in this influence, which indicates a similar distribution of the size groups of the evaluated subjects.

### Evaluation of district towns using 2 entry criteria (combinations D1-D10)

**Fig 16:**
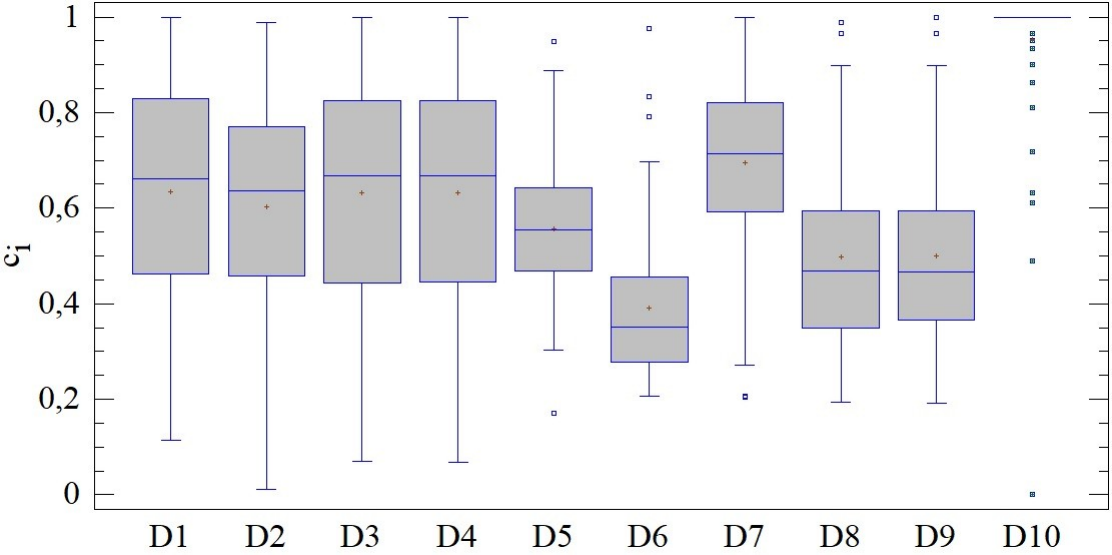
Results of SD-TOPSIS application ‐ combinations D1-D10.

Significant differences can be observed when comparing the results of the SD-TOPSIS technique including only 2 of the 5 entry criteria (combinations D1-D10). As can be identified from the figure below, differences can be found across all moment characteristics of level and position, variability or distribution shape. The variability expressed through the coefficient of variation ranges from 15.63% (combination D10) to 40.34% (combination D6), see Fig 16. In the case of the results with the lowest variability we also observe the highest concentration around the mean value (β = 25.30). These differences lead to the rejection of the assumption of homoscedasticity of the results, which cannot be confirmed (LE = 8.781; p < 0.01) due to significant differences in a total of 21 pairs.

Using the Kendall coefficient, with the exception of combination 10, a significant rank linear correlation with the results of combination A is confirmed. An interesting group consists of 4 combinations (D1, D2, D3, D4), where we can talk about an almost perfect rank correlation of their results with the results of combination A, i.e. a combination with 3 additional criteria (5 in total). In other cases, the strength of the correlation is 0.400, so it can be called weak or, in some cases, insignificant.

The agreement of the distribution functions of the results using 2 entry criteria (combinations D1-D10), with the results including all criteria in 3 combinations, was confirmed. The Kolmogorov-Smirnov test did not identify significant differences in pairs A-D1, A-D2, A-D3 and A-D4. In other cases, based on the results below, it is not possible to confirm the agreement in the structure of the results of the application of the SD-TOPSIS technique. Larger differences can be observed especially in the case of the D6 and D10 combinations. However, in the set of 10 combinations, it is also possible to find ones that copy the distribution of the results of combination A.

**Fig 17:**
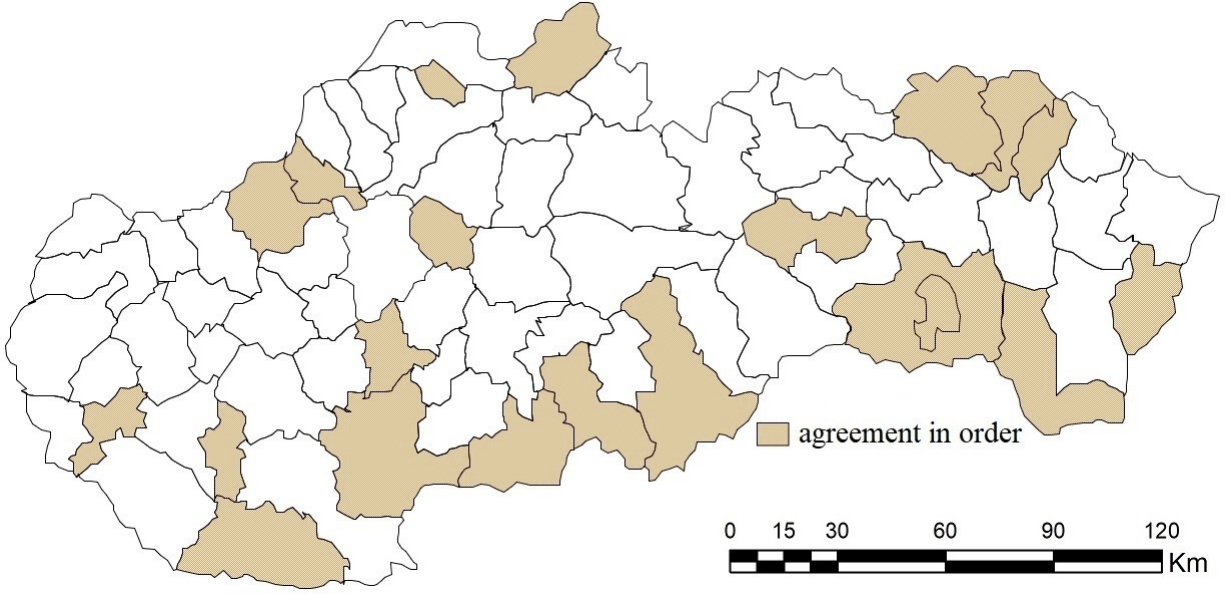
Agreement in the order of district towns in the results of the SD-TOPSIS application in space ‐ combination A vs. D2.

Based on the above, combinations D1-D10 can be divided into two groups. The first group (combinations D1-D4) match the ranking of combination A at the level of around 20%, while the best is combination D2 (28.16%), see Fig 17. Among the 20 district towns with the same order, it is possible to include, for example, Komárno, Levice, Senec and Šaľa. The second group of combinations achieves diametrically opposed results when comparing the ranking of district towns, while in the case of the combinations D6 and D7 not a single entity with the same ranking was found.

**Fig 18:**
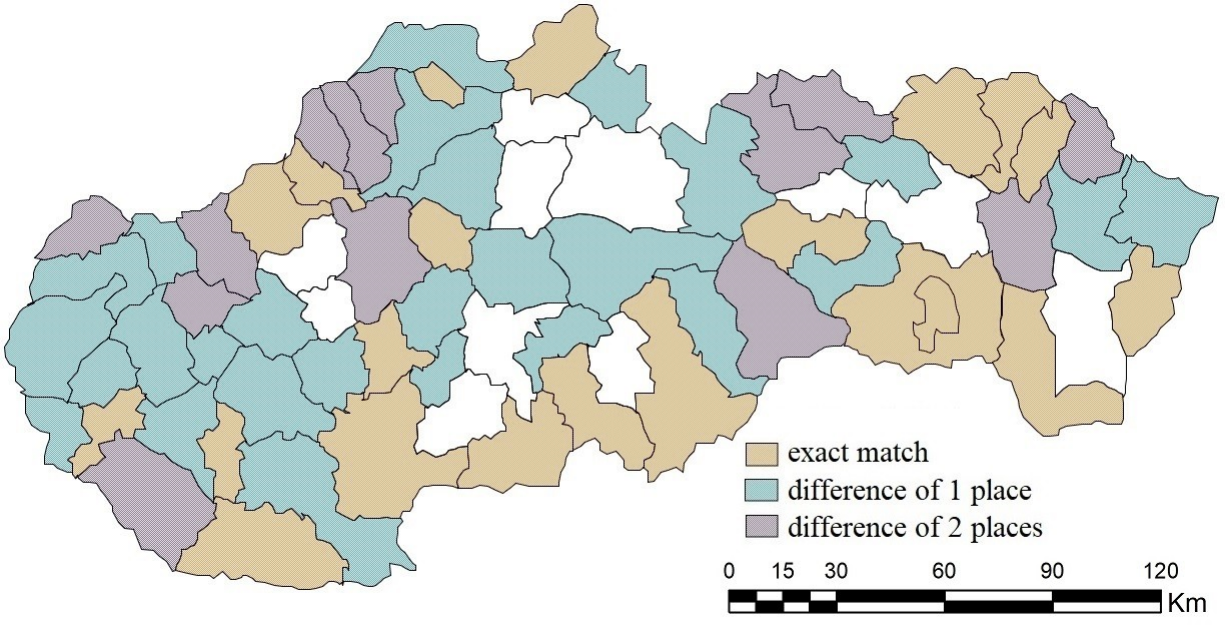
Agreement with tolerance in the order of district towns in the results of SD-TOPSIS application in space ‐ combination A vs. D2.

In the case of accepting a difference of 1 place in the order of evaluation by the SD-TOPSIS technique, the agreement of the order ranges from 45.07% to 67.60% in the case of combinations D1-D4. For combinations D5-D10, the maximum agreement is at the level of max. 11.26% (D6). It is the combination D2 that has the highest agreement in the order, with a tolerance of a difference of 2 places, namely 85.91% (see Fig 18). For combinations D1, D3 and D4, the agreement oscillates around 70% (D1–73.23%, D3–70.40%, D4–70.40%). On the contrary, with the combinations D6 and D10, even with this tolerance of 2 places, the agreement does not reach over 10% (D6–2.81%, D10–2.81%).

When evaluating the results of the SD-TOPSIS technique in the context of the size of the evaluated subject, the comparison is carried out by means of a regressor and a coefficient of determination; see the following table. The high informative value of the individual models remains, as well as the positive influence of the number of inhabitants on the evaluation results. Across the individual combinations, we do not record a significant difference in this influence, which is also documented by the stable level of the regressor.

### Comparison of the evaluation parameters of district towns across all combinations

**Fig 19:**
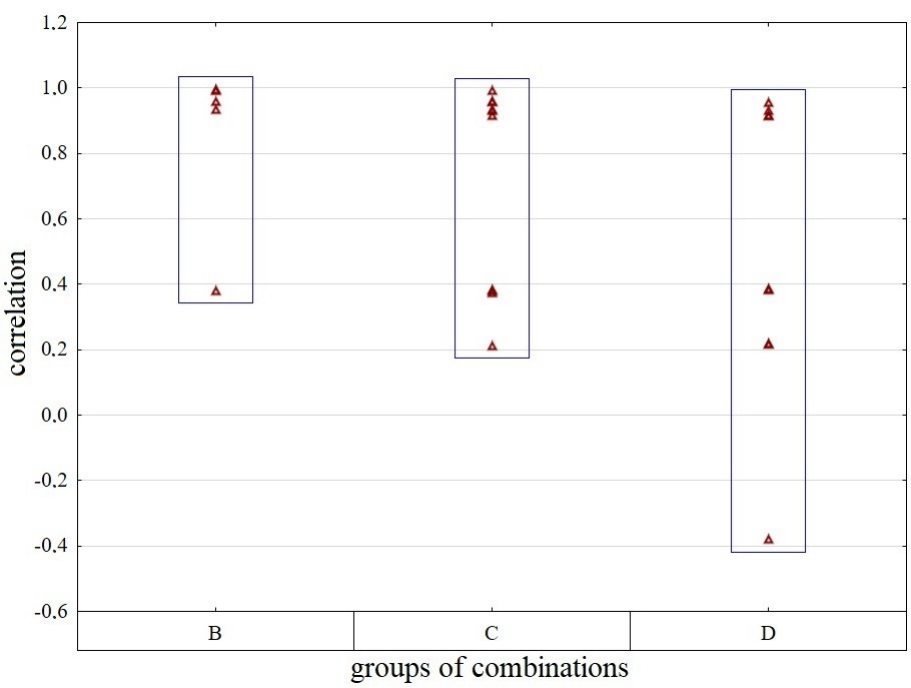
Comparison of rank correlation analysis results across groups of combinations - B1-B5 vs. C1-C10 vs. D1-D10.

The comparison of SD-TOPSIS multi-criteria evaluation parameters copies the structure of individual sub-analyses, with the intention of easier comparison or orientation in the text. The linear rank correlation of the results for combination A (5 input criteria) with the other results decreases in direct proportion to the decreasing number of criteria, see Fig 19. With 4 criteria, the significant correlation could be described as very strong or almost perfect only with 4 combinations. The average value is influenced by the result of the B5 combination out of the others (r_K_ = 0.381). With 3 criteria, we observe an average decrease of 6.40%, while with 5 combinations, the strength of the correlation is still above the level of 0.90. By reducing the number of criteria to 2, we still find 4 combinations with such a significant rank correlation (D1, D2, D3, D4). However, as the number of criteria decreases, the differences between the combinations increase, i.e. the relative and absolute variability of Kendall’s coefficient results increases.

As the number of entry criteria decreases, the number of confirmations of distribution function matching also decreases, but relatively slowly. Their agreement was confirmed in 80% of cases for B combinations, 60% of cases for C combinations and 40% of cases for D combinations. The structure of the results of the SD-TOPSIS technique therefore partially changes, but there are combinations where significant agreement remains.

**Fig 20:**
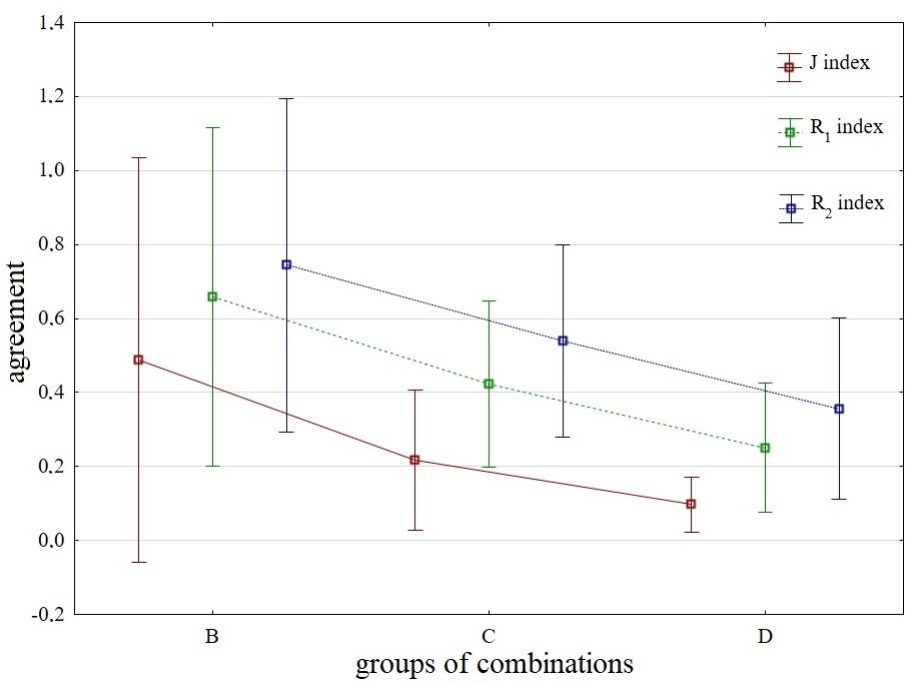
Comparison of agreement in the order based on the SD-TOPSIS technique across groups of combinations - B1-B5 vs. C1-C10 vs. D1-D10.

The average agreement in order quantified using the Jaccard index for B combinations reaches 48.73%, while it is significantly positively influenced by the results of combination B1 (97.18%) and combination B2 (94.36%). As the number of criteria decreases, the agreement decreases significantly, the exception being combination C1 with an agreement of 90.14%. In combinations with 2 criteria, the match is no longer acceptable, i.e. below 30% (in the case of combination D2). Accepting a difference of 1 place, we find in the case of combination B1 a complete agreement of the order, while the agreement in the case of combinations B2 and C1 exceeds 95%. With an accepted difference of 2 places in the order of district towns, we observe 100% agreement in the 2 mentioned combinations, while in the other 3 we reach the agreement level of min. 85% (specifically for combinations B4, C6 and D2). For detailed results, see Fig 20.

**Fig 21:**
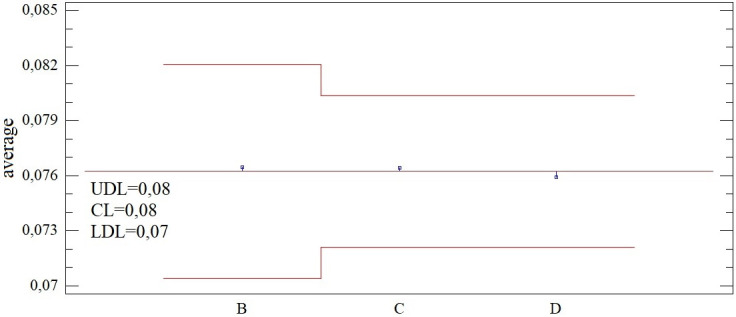
Comparison of the regressor within the regression model of SD-TOPSIS technique results across groups of combinations - B1-B5 vs. C1-C10 vs. D1-D10.

The above-mentioned differences do not affect the connection between the obtained results and the number of inhabitants of the district towns. The relationship of these variables across individual combinations proved to be highly relevant (the coefficient of determination does not fall below 93%) and constant. The rating of the SD-TOPSIS district town rises slightly with the growing number of its inhabitants, see Fig 21.

Based on the above-mentioned results of individual analyses, we can state that SD-TOPSIS is applicable with several combinations of entry criteria. Their suitability is determined by the degree of accepted deviation. In the case of an exact match in order, it is a combination of B1, B2 and possibly C1. The last-mentioned combination must be taken into account in the case of accepting a difference of one or two places. In the case of this combination of entry criteria, relevant results are achieved in comparison with combination A, which enables a reduction in the number of monitored criteria. Due to the identified combinations, criteria K4 ‐ Liabilities overdue to income and K5 ‐ Liabilities at least 60 days past due, could be considered.

### Comparison of overall results

Within the SD-TOPSIS combination, which determines the importance of entry criteria in the evaluation of district cities based on their absolute variability, it is possible to mark Tvrdošín (0.980 c_i_), followed by Poltár (0.928 c_i_), Nové Mesto nad Váhom (0.910 c_i_) and Topoľčany (0.859 c_i_) as the best-rated district towns. The results show no spatial correlation, with the best-rated district towns being located primarily in Central Slovakia, in the Žilina and Banská Bystrica self-governing regions. Based on the analyses carried out, we can state that SD-TOPSIS is applicable with a smaller number of criteria in the case of three combinations–B1, B2 and C1, in which criteria K4 ‐ Liabilities overdue to income and K5 ‐ Liabilities at least 60 days past due (simultaneously or separately), are absent. In the case of these combinations of entry criteria, relevant results are achieved compared to combination A, and are significantly correlated (B1: r_K_ = 0.970; p < 0.01; B2: r_K_ = 0.996; p < 0.01; C1: r_K_ = 0.992; p < 0.01) with confirmed agreements in the distribution functions (B1: K-S = 0.251; p = 1; B2: K-S = 0.167; p = 1; C1: K-S = 0.251; p = 1) and a similar positive effect of the number of inhabitants on overall results. The order of district towns in all three cases, with a tolerance of two differences, is 100% consistent with the order of SD-TOPSIS with the inclusion of all 5 criteria in the analysis (combination A).

**Fig 22:**
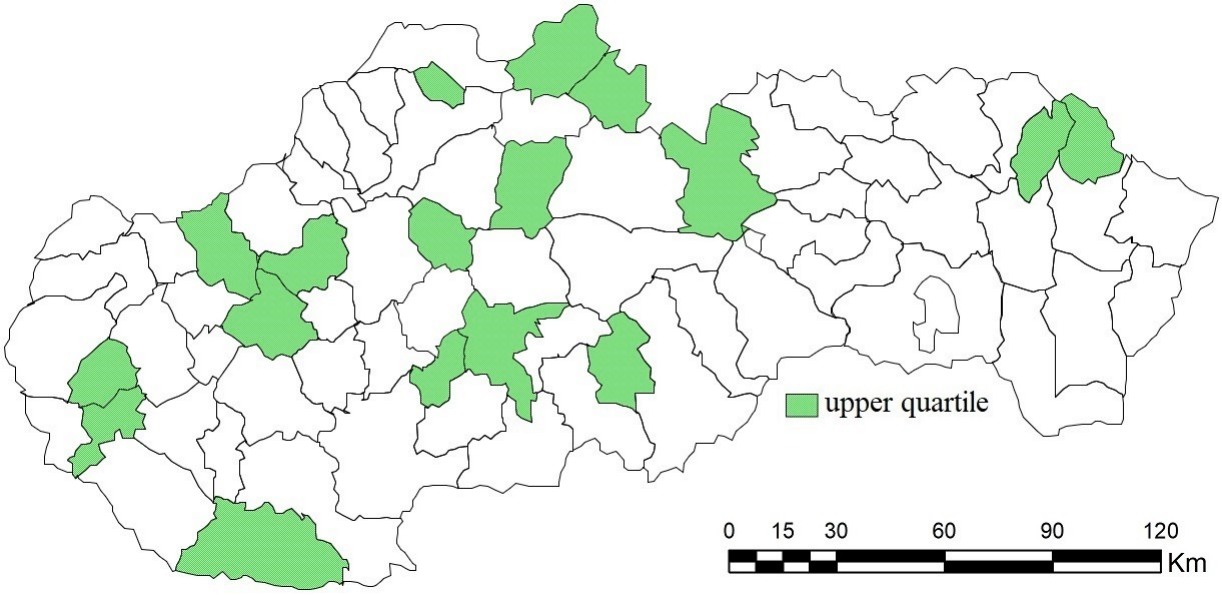
Upper quartile of district towns evaluated by the SD-TOPSIS technique ‐ combination of A, B1, B2 and C1.

In the case of the upper quartile of evaluated municipalities, the agreement of both combinations is complete, i.e. the district town, which was ranked among the top 25% for combinations A, was also ranked in this group for combinations B1. These entities include Banská Štiavnica, Pezinok, Senec and Stropkov. The majority part is located in Central and Western Slovakia, with the exception of Stropkov and Medzilaborce, located in the Prešov self-governing region, i.e. in Eastern Slovakia (see Fig 22).

**Fig 23:**
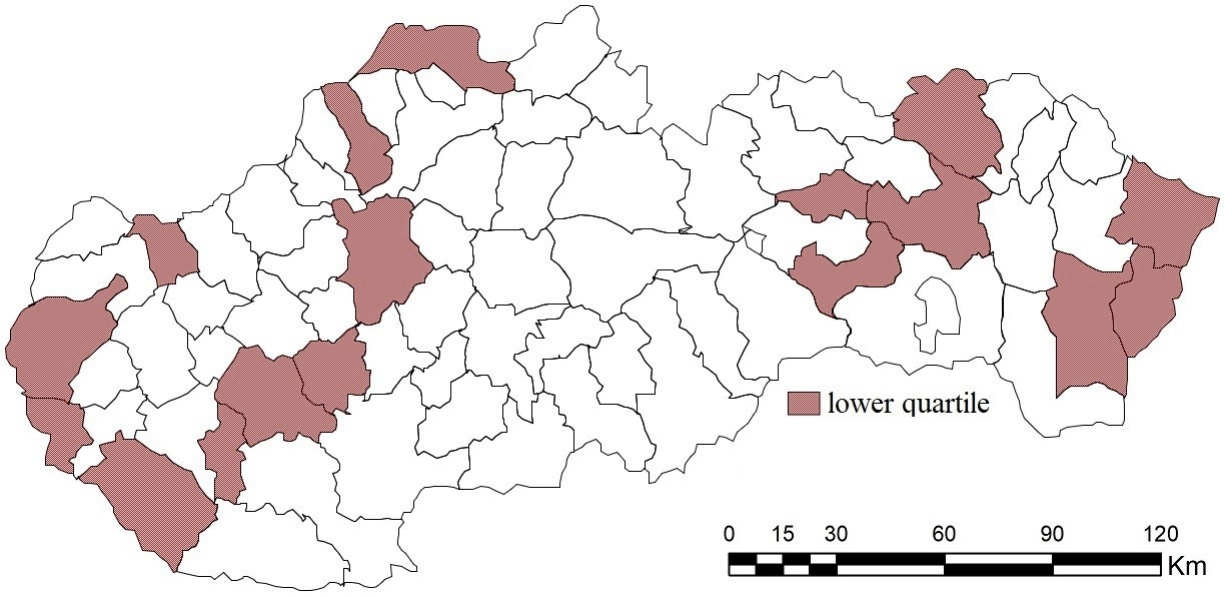
Lower quartile of district towns evaluated by the SD-TOPSIS technique ‐ combination of A, B1, B1 and C1.

A similar situation, with the agreement of the identified subjects, can also be observed in the case of the lower quartile, i.e. 25% of the worst-rated district towns. Each of the district towns located in this group with the A combinations is also located in it if criterion K4 or K5 is not included (i.e. combinations B1, B2 and C1). Such entities include Čadca, Sobrance, Snina and Nitra. From the point of view of spatial distribution, we observe the dominant representation of Eastern Slovakia (7); see the following figure/map (Fig 23).

In the case of the SD-TOPSIS technique, combinations B1, B2 and C2, with the absence of criteria K4 and K5, are full replacements.

As a result, the multi-criteria evaluation of territorial self-government subjects (in our case, district towns) proved to be highly applicable. However, the result itself is largely determined by the structure and number of entry criteria. Based on the performed analyses described in the previous sections, we can state that there are significant differences resulting from the reduction in the number of entry criteria. Each such reduction had an impact on the overall results, but it is possible to find combinations that deviate from this conclusion (combinations B1, B2 and C2). They could be the subject of further research, or verification on another research sample.

## Conclusions

As a result, the multi-criteria assessment of local government entities using TOPSIS method (in our case, district towns in Slovakia) proved to be highly applicable. However, the result itself is largely determined by the structure and number of criteria. On the basis of the analyses, we can observe significant differences resulting from a reduction in the number of criteria. Based on the results of our own research, we can conclude that, each criteria reduction had an impact on the overall results, but it is possible to find combinations that escape this conclusion (combinations B1, B2 and C2). These are the ones that could be the subject of further research or verification on a different research sample.

Several applications of MCDM methods in local government conditions work with a heterogeneous number of criteria [4, 5, 8, 15], especially in Slovakia [10–15]. However, none of these applications think about the number of criteria used. The novelty of the research findings is demonstrated on two levels:

- the use of 5 specific criteria that have not yet been processed using MCDM analysis methods,
- the introduction of the idea of reducing the number of criteria used in order to simplify the multi-criteria models used so far.

A similar approach to identifying opportunities for reducing the number of criteria can be applied across the private and public sectors. We see an opportunity in financial analysis, HR management, management assessment, logistics and emphasis on supplier selection, customer behavior, and others.

The results obtained should be seen with the following limitations:

- the simplest method (or one of the simplest methods) for determining the importance of criteria was used; it is based on the variability of the criteria in absolute terms,
- approaches to determining the importance of criteria are innumerable [41, 65–82], and different results can be expected with their application,
- it is the same with MCDM methods, the TOPSIS method is used, the use of which in the conditions of (Slovak) territorial self-government has been proven [4, 5, 8, 10–15].
